# Nutritional stress reprograms dedifferention in glioblastoma multiforme driven by PTEN/Wnt/Hedgehog axis: a stochastic model of cancer stem cells

**DOI:** 10.1038/s41420-018-0126-6

**Published:** 2018-12-05

**Authors:** Susmita Mondal, Kaushik Bhattacharya, Chitra Mandal

**Affiliations:** 1 Cancer Biology and Inflammatory Disorder Division, Council of Scientific and Industrial Research (CSIR)-Indian Institute of Chemical Biology (IICB), 4, Raja S.C. Mullick Road, Jadavpur, Kolkata, 700032 India; 2 Present Address: Département de Biologie Cellulaire, Université de Genève, Sciences III, 30 Quai Ernest-Ansermet, 1211 Genève 4, Switzerland

## Abstract

The emergence and maintenance of cancer stem-like cells (CSCs) are usually governed by tumor niche. Tumor niche always provides metabolic challenges to cancer cells and CSCs mostly because of tissue hypoxia. However, the role of micro-environmental nutritional stress (NS) in dedifferentiation of cancer cells is poorly defined. Here, we developed a stochastic model of CSCs by gradual nutritional deprivation in glioblastoma multiforme (GBM) cells used as a model system. Nutritional deprivation induced enhanced expression of glioblastoma stem-like cells (GSCs)-specific biomarkers with higher invasive and angiogenic properties. This NS-induced cells showed higher xenobiotic efflux ability, and hence exhibit resistance to multiple anticancer drugs. In the molecular level, such NS activated Wnt and Hedgehog (Hh) signaling pathways by stabilizing β-catenin and Gli1, respectively, through modulation of GSK3β/AKT axis. GBM-specific PTEN (phosphatase and tensin homolog) mutation contributed to better phenoconversion toward GSCs. Knocking down of PTEN coupled with NS induction enhanced neurosphere formation, GSC-specific biomarker expressions, and activation of Wnt/Hh signaling. Thus, such an in-depth understanding of dedifferentiation of GBM cells to GSCs under NS suggested that targeting Wnt/Hh signaling possibly be a better therapeutic approach.

## Introduction

Tumors possess similar hierarchy like normal tissues^1,2^. Their heterogeneity is maintained by a small subset of cell population defined as cancer stem-like cells (CSCs)^3,4^. CSCs undergo asymmetric division and are responsible for the propagation, invasion, metastasis, and recurrence^5^. Deregulated Wnt/β-catenin and Hedgehog (Hh) signaling pathways promote tumor progression by sustaining CSCs^6^.

Initial mutations in normal stem cells may generate CSCs that possess the attribute of self-renewal and multipotency. Alternatively, CSCs are generated from differentiated cancer cells through mutations causing dedifferentiation^7^. A stochastic model hypothesizes that each cell in a tumor mass has the potential for propagating tumor, whereas the hierarchy model suggests that only a few cells with oncogenic potential can proliferate and differentiate^8,9^.

Tumor niche with hypoxia, less nutrients, and low pH leads to an altered metabolic and physicochemical milieu^10,11^. Reciprocal interactions between cancer cells to its microenvironment play a crucial role in tumor progression^12^. Continually changing microenvironment empowers the adaptive nature of these cells, leading to progression, invasion, and metastasis. Micro-environmental stress-driven selection forces might be responsible for behaving like CSCs^13^.

Glioblastoma (grade IV) is an aggressive primary malignant brain tumor with dreadful prognosis^14,15^. Inactivation of PTEN (phosphatase and tensin homolog), a tumor suppressor protein, is associated with glioblastoma multiforme (GBM), and correlated with increased malignancy and higher mortality^16,17^.

However, the role of nutrient deprivation toward CSCs is still unclear. Here, we aimed to decipher the emergence and maintenance of glioblastoma stem-like cells (GSCs) upon nutritional stress (NS). We provide evidences for the NS-mediated stochastic emergence of GBM stem-like cells (GSCs), and this phenoconversion is mainly guided through higher Wnt/Hh activities. Furthermore, PTEN mutation assists in the transition from differentiated GBM cells to GSCs by modulating Wnt/β-catenin and Gli1 activity via AKT/GSK3β signaling cascade. Therefore, inhibition of Wnt/Hh signaling molecules could be an alternative approach to manage GSCs.

## Results

### NS induces a phenotypic transition from differentiated GBM cells to GSCs

**Fig. 1:**
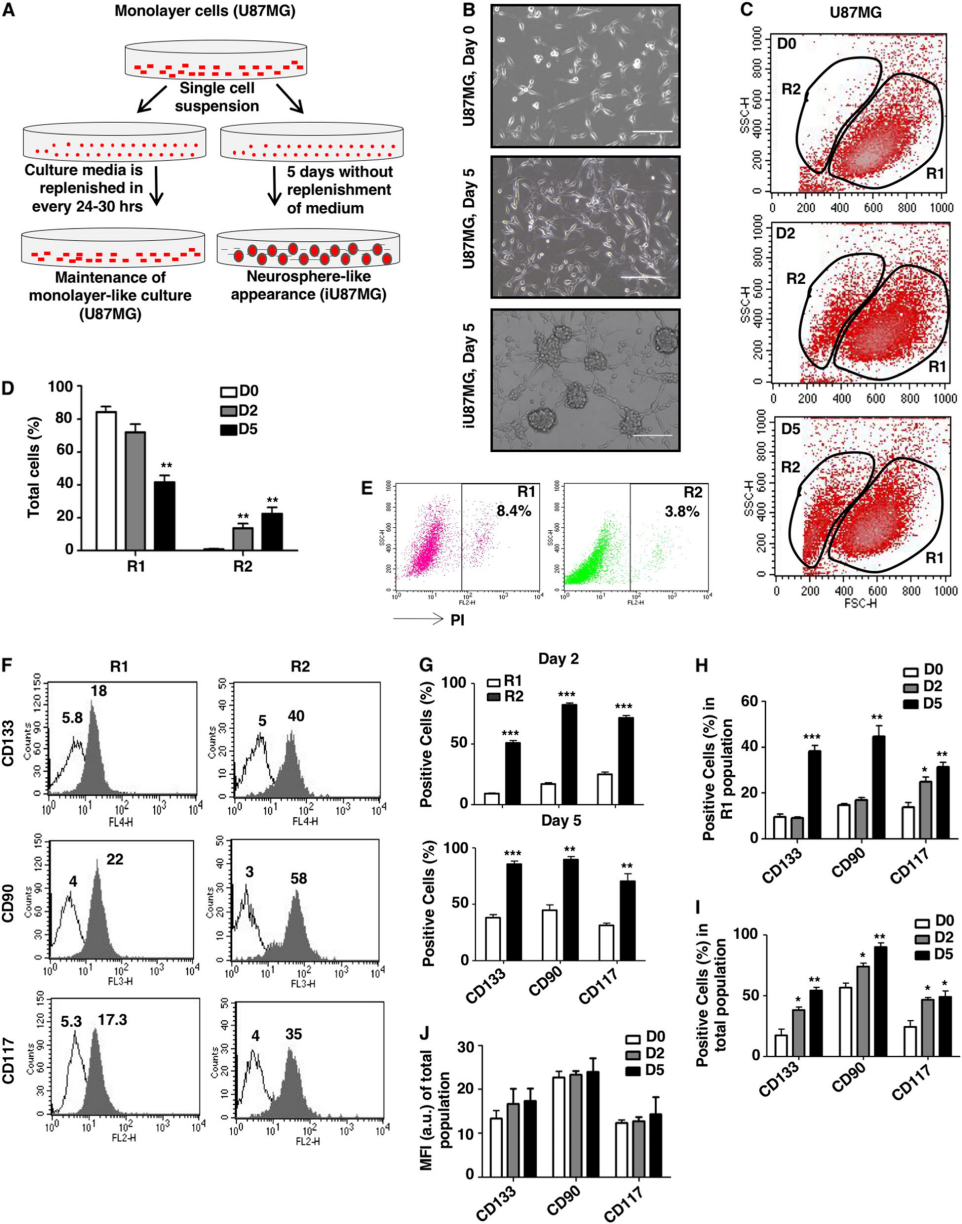
A phenotypic transition of GBM cells upon nutritional stress. **a** Schematic representation of the workflow of gradual nutrient depletion. Single-cell suspensions were made and cells (5 × 10^5^) were seeded in a six-well plate in 2 ml of IMDM with 10% FBS and cultured for 5 days in the CO_2_ incubator without replenishment of fresh medium. **b** Representative phase-contrast images with ×10 magnification, showing the sphere-like appearance of GBM cells (iU87MG) upon 5 days of nutritional stress, whereas there was no sphere formation upon continually changing the medium. **c** Shifting of cell sizes toward smaller-sized population (gated as R2) of a fraction of the remaining population (gated as R1) at day zero (D0), 2 (D2), and 5 (D5) as assessed by flow cytometry. **d** Graphical representation of the percentage of cells shifting toward smaller-sized R2 population at D0, D2, and D5. **e** Propidium iodide (PI) staining showed a very low percentage of apoptotic cells in the R1 and R2 population after 5 days of nutritional stress. **f** Representative histogram plots showing differential expression of GSC markers (CD133-APC, CD90-PECy5, and CD117-PE) in the R1 and R2 population as represented by mean fluorescence intensity (MFI) at day 5 of nutritional stress. White peaks represent autofluorescence of the cells in the R1 and R2 populations, and gray peaks represent MFI of GSCs markers (CD133-APC, CD90-PECy5, and CD117-PE) in the R1 and R2 populations. Values on the peak represent the MFI values of the markers. **g** Cell surface staining followed by flow cytometry analysis determined the percentage of CD133-APC-, CD90-PECy5-, and CD117-PE-positive cells in the R1 and R2 population at day 2 and 5 of nutritional stress. **h** Cell surface staining at different time points (0, 2, and 5 days) of nutritional stress followed by flow cytometry analysis determined time-dependent enhancement in the expression of GSCs markers in the R1 population. **i** Flow cytometric analysis revealed time-dependent stochastic increase of the number of CD133-APC-, CD90-PECy5-, and CD117-PE-positive cells in the total population under nutritional stress. **j** Flow cytometric analysis showed a slight increase in the MFI of GSCs makers in the total population at different time points due to nutritional stress. The results represent mean ± SD from three independent experiments

The tumor microenvironment has tumor-promoting functions in different stages of oncogenesis^18^. Nutritional depletion-mediated metabolic stresses are usually experienced by CSCs at their niche^19^. To understand the impact of NS in the formation of GSCs from differentiated cells, U87MG cells were cultured in complete growth medium for 5 days without replenishing fresh medium to mimic the microenvironment of tumor niche (Fig. 1a).

A considerable fraction of the NS-induced U87MG cells (iU87MG) showed neurosphere-like appearance after 5 days compared to media-replenished condition (Fig. 1b). This phenotypic change was monitored by flow cytometry, which revealed a gradual generation of a new smaller-sized cell population (R2) with lower forward scattering in iU87MG (Fig. 1c). The parental cell was gated as R1. The number of cells was increased gradually in the R2 population upon NS (Fig. 1d). The viability of these cells was reflected by lower propidium iodide (PI) positivity (Fig. 1e). These initial observations led us to think that NS possibly induced dedifferentiation of the differentiated cancer cells to exhibit CSC-like phenotypes.

### NS induces GSC-like cell surface marker expressions in favor of stochastic dedifferentiation

To establish our hypothesis, we checked the expressions of conventional GSCs markers (CD133, CD90, and CD117) on NS-induced cells. We observed an increase in mean fluorescence intensity (MFI) of GSC markers and a higher number of marker-positive cells in the R2 population than in the R1 population at 2nd and 5th days (Fig. 1f, g). A significant gradual increase in expressions of all three markers was also observed in the R1 population (Fig. 1h). Hence, both the R1 and R2 populations showed a stochastic adaptation upon NS induction. Subsequently, we analyzed the R1 and R2 combined populations to determine such stochastic changes for further validation of our hypothesis. We observed an overall increase in the GSC markers from 0 to 5 days after NS induction. NS-induced cells exhibited an ~3-fold increase in CD133^+^ and ~2-fold enhancement in both CD90^+^ and CD117^+^ cells compared to the parental population (Fig. 1i). However, there were no statistically significant changes in the MFIs in iU87MG, which signify that NS induction leads to the conversion of U87MG cells to its GSCs state instead of increasing the stemness of GSCs already present in the population (Fig. 1j).

### NS-induced GBM cells exhibit GSC-like molecular and functional phenotypes

**Fig. 2:**
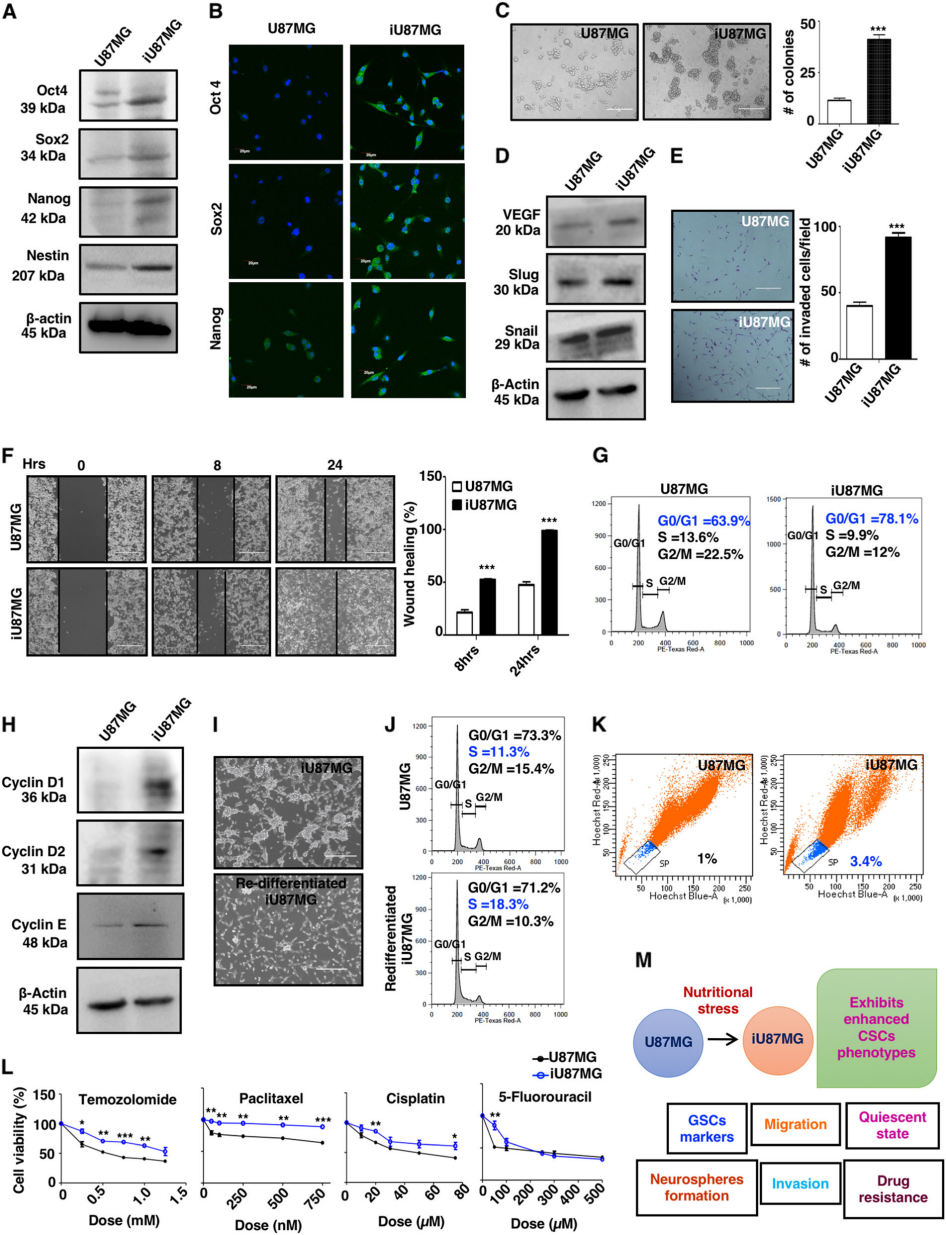
Functional and quiescent properties of NS-induced cells (iU87MG). **a** Cell lysates were prepared from U87MG and iU87MG cells and resolved by sodium dodecyl sulfate-polyacrylamide gel electrophoresis (SDS-PAGE), and then analyzed by western blot analysis with the specified antibodies. Representative immunoblots show an increased level of pluripotent stem cell markers (Oct4, Sox2, and Nanog) and Nestin at the protein level in iU87MG compared to U87MG. β-Actin served as a loading control. **b** U87MG and iU87MG (1 × 10^4^) cells were adhered on poly-l-lysine-coated coverslip and processed for immunocytochemistry with Oct4, Sox2, and Nanog primary antibodies followed by incubation with Alexa Flour 488-conjugated secondary antibodies. Representative confocal images merged with DAPI staining represented the enhanced expression of Oct4, Sox2, and Nanog in iU87MG compared to U87MG. **c** Cells were trypsinized, resuspended, counted, and seeded in stem cell-specific medium in ultra-low-attachment plates. Representative phase-contrast images demonstrated higher neurospheres formation after 72 h by iU87MG compared to U87MG. Bar graph represents quantitative measurements of the number of neurospheres per field (*n* = 7). **d** Western blots analysis from cell lysates of U87MG and iU87MG showing enhanced expression of Slug, Snail, and VEGF at the protein levels in iU87MG. β-Actin served as a loading control. **e** Transwell invasion assay showing higher invasive property of iU87MG. Representative phase-contrast images of invaded cells to the lower surface of the insert after 36 h. Cells were stained with crystal violet and a number of cells were counted (*n* = 9). **f** U87MG and iU87MG cells were seeded in six-well plates in serum-free medium. Scratch was made using the 10-µl tip in each well. Representative phase-contrast images showed cellular migration and filling of the gap after 8 and 24 h. The average wound was calculated and represented as the percentage of wound healing (*n* = 5). **g** U87MG and iU87MG cells were trypsinized and single cells were made and processed for cell cycle analysis. Representative histogram plots demonstrating restriction at the G0/G1 phase of iU87MG as compared to U87MG. **h** Representative immunoblots showing higher expression of Cyclin D1, Cyclin D2, and Cyclin E at the protein level in iU87MG compared to U87MG. β-actin was used as a loading control. **i** Representative phase-contrast images of re-differentiated iU87MG showing differentiated morphology in the fresh medium. Images were taken after 24 h of re-seeding. **j** Cell cycle analysis of U87MG and re-differentiated iU87MG showing similar trends and progression into the S phase after 24 h of re-seeding in the fresh medium. **k** Hoechst side population (SP) assay showing enhanced Hoechst-negative side population in iU87MG denoting enhanced drug efflux potentiality of these cells. **l** U87MG and iU87MG cells were treated with temozolomide, paclitaxel, 5-fluorouracil, and cisplatin separately. Cell viability assay of treated cells showed enhanced drug resistance in iU87MG than U87MG in a dose-dependent manner. The significance levels were shown between two groups using asterisks in each dose. **m** Schematic representation of nutritional stress-mediated stochastic emergence of CSC phenotype in GBM. Results represent mean ± SD from three independent experiments

Next, we observed enhanced expression of pluripotent stemness markers Oct4, Sox2, and Nanog both in messenger RNA (mRNA) and protein levels in iU87MG (Figure. S1A and Fig. 2a) along with a GSC-specific intracellular marker, Nestin (Fig. 2a). ALDH1A1, a common marker for CSCs, was enhanced at the genetic level (Figure S1A). Increased expressions and nuclear localizations of Oct4, Sox2 and Nanog were also observed by confocal microscopy (Fig. 2b).

Subsequently, both parental and iU87MG cells were plated in a stem cell-specific medium in ultra-low-attachment plates for 72 h for neurosphere formation, which revealed an ~4-fold increment in the number and larger neurosphere formation by iU87MG (Fig. 2c). This iU87MG also exhibited enhanced ability to form 3D neurospheres on Matrigel, an artificial extracellular matrix (Figure S1B).

CSCs are mainly responsible for the invasive and migratory properties of cancer^20,21,^ and Slug/Snail are two key molecules, whereas vascular endothelial growth factor (VEGF) is involved in angiogenesis. iU87MG exhibited an increased level of Slug, Snail and VEGF both in mRNA and protein levels (Figure S1C and Fig. 2d). These observations were further validated by in vitro invasion assay using Matrigel-coated insert systems. iU87MG exhibited higher invasion than U87MG within 36 h (Fig. 2e).

The angiogenic property of iU87MG was demonstrated by the connective tube formation assay. They exhibited more thread-like connections between themselves (Figure S1D). Moreover, iU87MG showed higher wound-healing rate in scratch-wound assay within 24 h, whereas U87MG cannot fill the gap as efficiently (Fig. 2f).

### NS-induced iU87MG cells are quiescent in cell cycle progression like CSCs

CSCs are usually quiescent in nature with the characteristic of more cells in the G0/G1 phase^22^. iU87MG exhibited significantly more cells at the G0/G1 phase (Fig. 2g) and enhanced expression of a few G0/G1-phase regulatory molecules (Cyclin D1, Cyclin D2 and Cyclin E) both in mRNA and protein levels (Figure S1E and Fig. 2h). Moreover, iU87MG exhibited re-differentiation properties upon splitting with the fresh complete medium, as assessed by their morphology similar to U87MG (Fig. 2i), and cell cycle pattern showing more cells in the S phase after 24 h (Fig. 2j).

### NS-induced cells are more resistant to cancer therapeutics, and efficient to efflux xenobiotics

CSCs are more resistant to chemotherapy due to its efficient efflux machinery^23,24^. Accordingly, we measured drug-resistant cells as evaluated by Hoechst-negative population, referred as side population. We observed an ~3-fold increase in the side population of iU87MG, suggesting more xenobiotics efflux potential and probable drug resistance phenotype (Fig. 2k). To understand drug-resistant property, we treated both the cells with four anticancer drugs (paclitaxel, temozolomide, 5-fluorouracil and cisplatin), and observed a significant less death in iU87MG (Fig. 2l), indicating the role of NS in inducing more drug-resistant properties. So far, we have validated that NS-induced cells exhibit all the key attributes of CSCs (Fig. 2m).

### NS-induced cells exhibit better survivability upon subsequent metabolic stresses

CSCs usually suffer severe combined metabolic stresses in the core of tumor. However, they are capable of modulating themselves for the adaptation to overcome these stresses for their survival^25,26^.

**Fig. 3:**
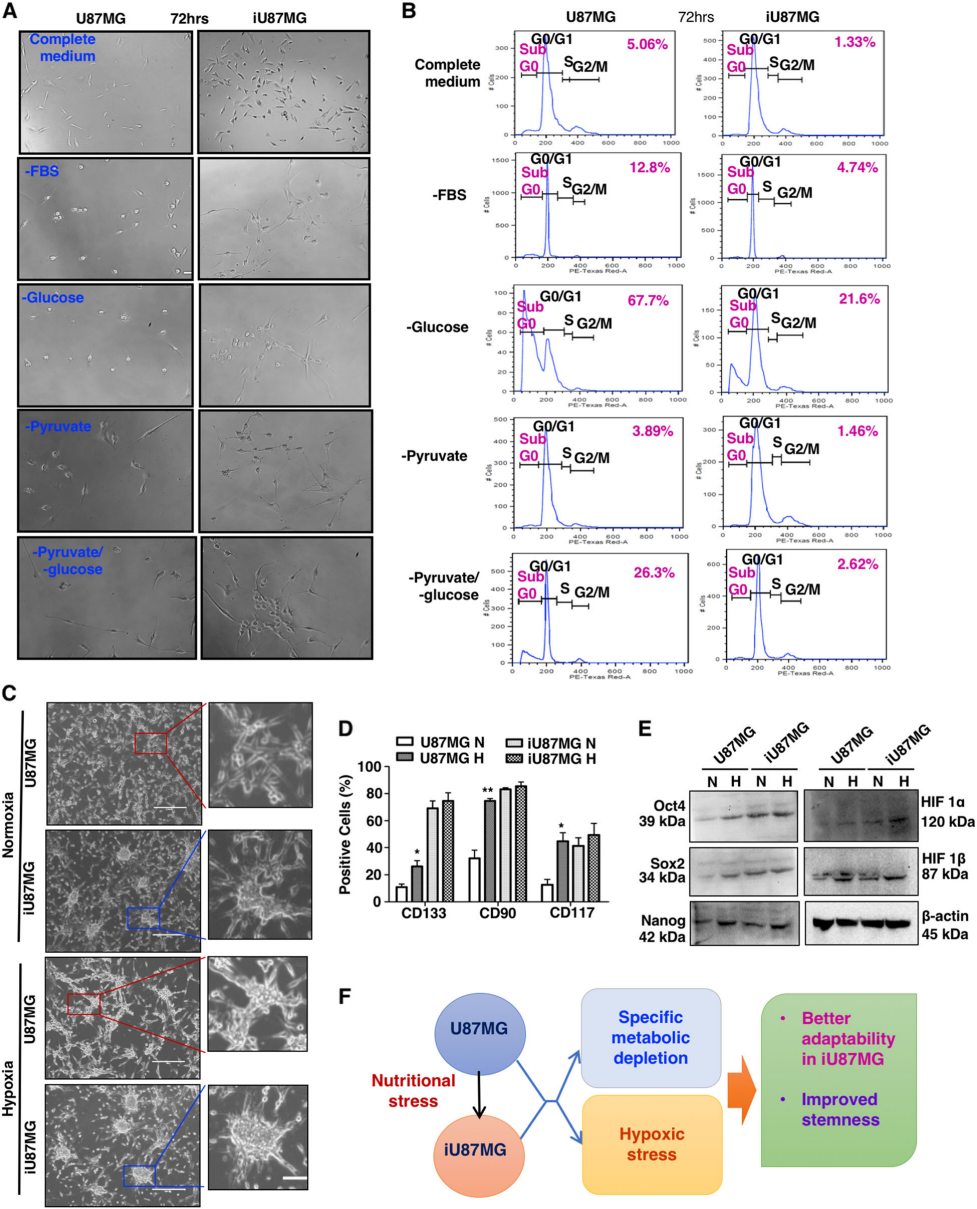
Better metabolic adaptations by iU87MG. **a** U87MG and iU87MG cells were kept in selective metabolic stress conditions separately, such as depletion of serum, glucose, and pyruvate, along with glucose and pyruvate together for 72 h. Representative phase-contrast images showing better survivability in iU87MG in all the deprived conditions compared to U87MG. **b** Cell cycle analysis of U87MG and iU87MG kept under selective metabolic-depleted conditions demonstrated higher cell death in U87MG, indicating better metabolic stress adaptability of iU87MG cells. **c** Representative phase-contrast images showing neurospheres-like morphology upon the hypoxic condition in U87MG and both in normoxic and hypoxic conditions in iU87MG. **d** The expression profile of GSCs markers (CD133-APC, CD90-PECy5, and CD117-PE) in U87MG and iU87MG under normoxic (N) and hypoxic (H) conditions as assessed by flow cytometry. **e** Representative immunoblots representing the status of pluripotent stem cell markers (Oct4, Sox2, and Nanog), and HIF-1α and HIF-1β upon additional hypoxic stress in both U87MG and iU87MG. β-actin served as a loading control. **f** Schematic representation of metabolic and hypoxic stress adaptation of iU87MG. Results represent mean ± SD from three independent experiments

Accordingly, to check the effect of metabolite-deprived conditions, we exposed iU87MG and U87MG in the medium deplete of glucose, pyruvate, and glucose and pyruvate together, as well as the serum to deprive growth factors. As expected, cells grown in the complete medium showed better morphology than metabolically stressed conditions. However, iU87MG exhibited better survivability in all these deprived conditions, as depicted by phase-contrast images after 72 h (Fig. 3a) and lower sub-G0 population (Fig. 3b). However, glucose deprivation caused the maximum cell death, suggesting their highest dependency on glycolysis and cannot withstand such stress.

To add another level of micro-environmental stress, cells were cultured in hypoxic condition for 24 h, as ensured by enhanced expression of hypoxia-inducible factor-1α (HIF-1α) and HIF-1β. Hypoxia-induced U87MG showed neurosphere-like appearances and increased GSC marker expressions as expected. However, iU87MG exhibited more and larger neurosphere-like morphology under such conditions (Fig. 3c), and slightly higher expression of CD133, CD117 and CD90 compared to normoxic condition (Fig. 3d). Furthermore, hypoxia-induced iU87MG showed enhanced expressions of Oct4, Sox2 and Nanog in protein levels (Fig. 3e). Therefore, we may conclude that NS-induced cells showed better adaptability upon additional metabolic stresses (Fig. 3f).

### NS-induced GSCs exhibit hyperactivation of early stem cell signaling pathways

**Fig. 4:**
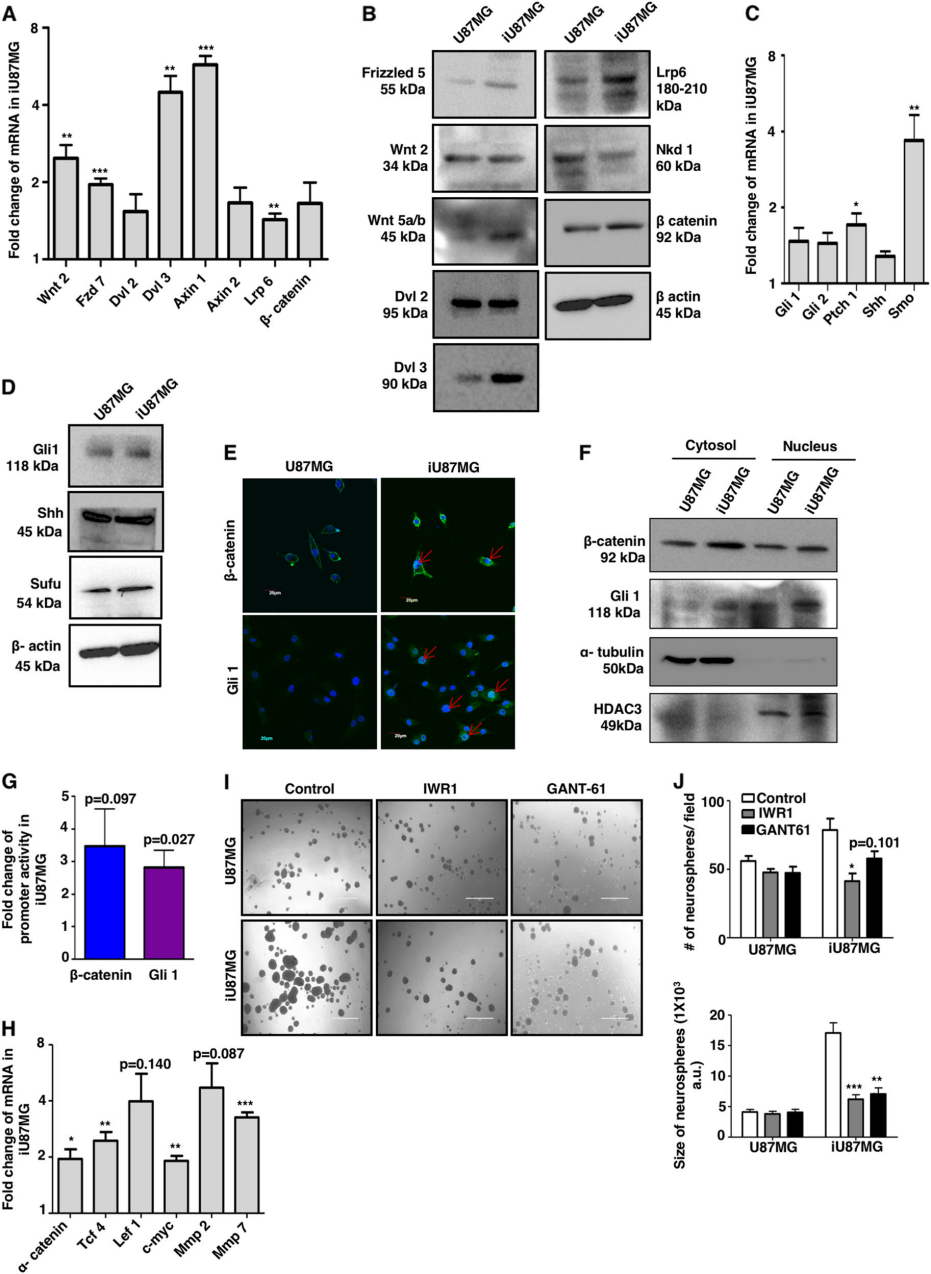
Involvement of stem cell regulatory pathways in NS-induced cells (iU87MG). **a** Expression of canonical Wnt/β-catenin pathway genes in iU87MG compared to U87MG was assessed by real-time qPCR. Values are normalized against 18S rRNA expression. **b** Representative immunoblots analysis showing enhanced expression of Wnt/β-catenin pathway proteins in iU87MG compared to U87MG. **c** mRNA expression of sonic Hedgehog pathway genes showing upregulation in iU87MG compared to U87MG as assessed by real-time qPCR. Values are normalized against 18S rRNA. **d** Western blots analysis showing enhanced expression of sonic Hedgehog pathway proteins in iU87MG compared to U87MG. **e** Representative confocal images merged with DAPI staining represented the enhanced cellular accumulation and nuclear localization of β-catenin and Gli1 transcription regulators in iU87MG compared to U87MG. **f** Western blots of cytosolic and nuclear fractions from U87MG and iU87MG showing enhanced nuclear localization of β-catenin and Gli1 in iU87MG. α-Tubulin and HDAC3 served as internal controls for cytosol and nucleus, respectively. **g** Graphical representation of fold change of relative promoter activity of β-catenin and Gli1 transcription regulators, as assessed by luciferase assay in iU87MG compared to U87MG. **h** Real-time qPCR analysis showing higher expression of a few target genes of β-catenin and Gli1 at the mRNA level in iU87MG compared to U87MG. **i** Representative phase-contrast images showing the effect of Wnt/β-catenin and sonic Hedgehog pathway inhibitors (IWR-1 and GANT-61, respectively) in the neurosphere formation in both U87MG and iU87MG. **j** Quantitative representation of number and size of neurospheres upon inhibition of Wnt/β-catenin and sonic Hedgehog pathways by IWR-1 and GANT-61, respectively, in both U87MG and iU87MG. Results represent mean ± SD from three independent experiments

Next, we investigated whether activation of canonical Wnt signaling is involved in phenotypic transition after NS induction. We found significantly enhanced expression of Wnt2, Frizzled-7, Disheveled-3, Axin1, Lrp6 and β-catenin in mRNA levels (Fig. 4a), and some of these molecules even in protein levels in iU87MG (Fig. 4a, b). Furthermore, Naked-1, a negative regulator of Wnt signaling, was also decreased upon NS. In contrast, we found a minute increase of Gli1, Gli2, Patch1, Smo, Shh and Sufu involved in Hh pathway both in mRNA and protein levels (Fig. 4c, d).

Conversely, the Notch pathway, the third major CSC-related signaling pathway, was downregulated in iU87MG (Figure S2A), indicating that NS-induced dedifferentiation may be mainly linked with Wnt and Hh signaling pathways, which possibly is responsible for triggering developmental reprogramming in cancer microenvironment.

β-Catenin and Gli1 are the two master transcriptional regulators of Wnt and Hh signaling pathways, respectively. Enhanced cellular accumulation and nuclear localization of β-catenin and Gli1 were observed by confocal microscopy and western blot analysis in iU87MG (Fig. 4e, f). Furthermore, luciferase reporter assay revealed a higher promoter activity of β-catenin and Gli1, suggesting enhancement of Wnt and Hh pathways upon NS in iU87MG (Fig. 4g).

To further validate the functionality of these transcriptional regulators, we checked the status of a few common target genes. iU87MG showed higher expression of α-catenin, TCF4, lef1, c-myc, MMP2 and MMP7 in mRNA, and TCF1 and LEF1 in protein levels (Fig. 4h and Figure S2B).

To understand the dependency on Wnt and Hh signaling of NS-mediated dedifferentiation of U87MG, both the cells were treated separately with IWR-1 and GANT-61, inhibitors of respective pathways, and monitored their neurosphere formation capability. iU87MG showed a marked reduction in the neurosphere formation, whereas no significant changes were observed in U87MG (Fig. 4i, j). Next, U87MG cells were pre-treated with these inhibitors and subsequently exposed to NS for 5 days, and further cultured in neurosphere-forming medium. We observed less number and smaller neurosphere formation in the presence of these inhibitors, suggesting a good correlation between Wnt/Hh activity, and NS-induced dedifferentiation in glioblastoma (Figure S2C).

### Activation of β-catenin and Gli1 in NS-induced GSCs is negatively regulated by GSK3β

**Fig. 5:**
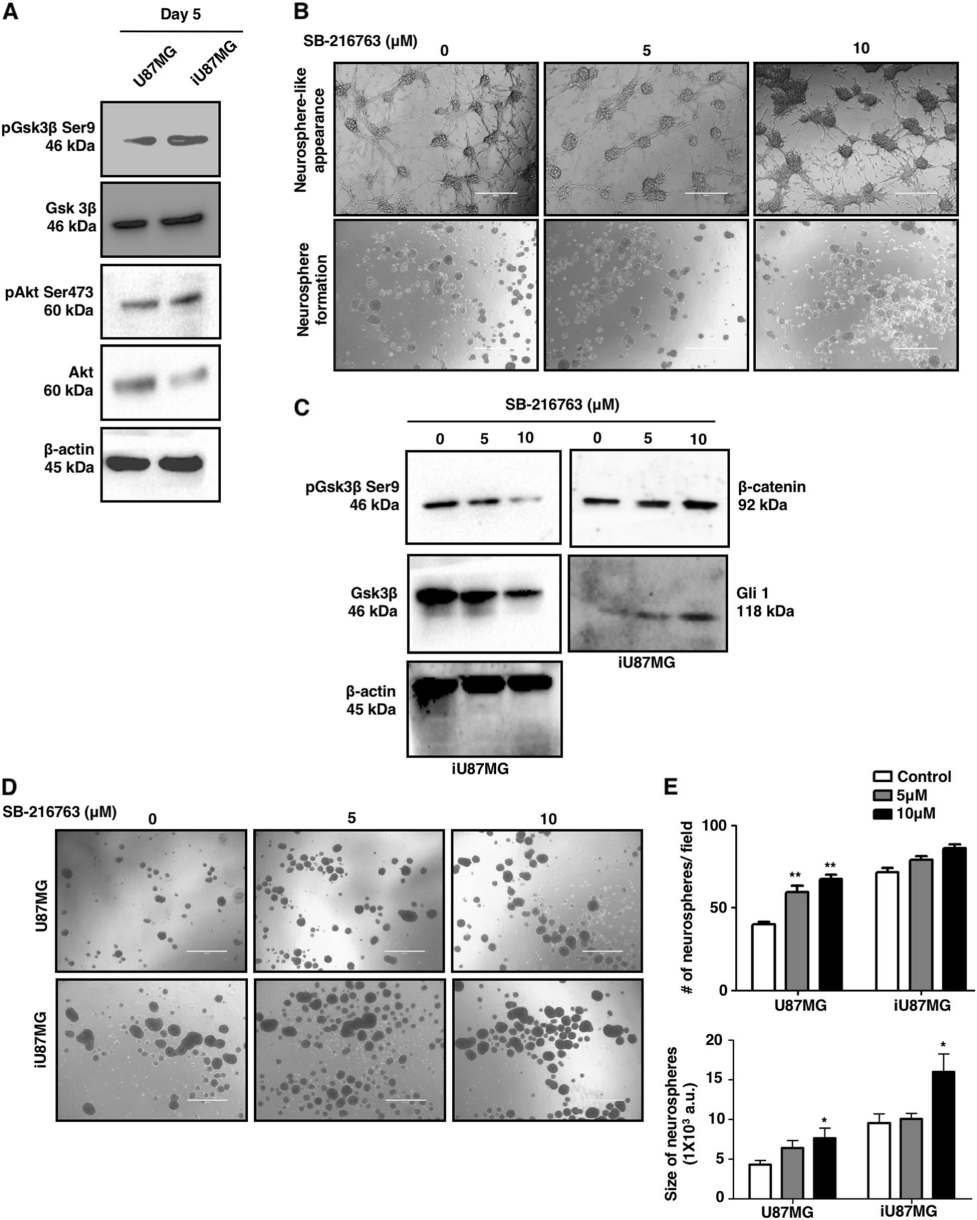
GSK3β-mediated activation of Wnt/β-catenin and sonic Hedgehog pathway in NS-induced cells. **a** Representative immunoblots showing higher GSK3β phosphorylation at the Ser9 position leading to lower GSK3β activity in iU87MG compared to U87MG, which is mediated by higher AKT phosphorylation at Ser473. β-Actin was used as a loading control. **b** Phase-contrast images showing the larger neurosphere-like appearance of NS-induced cells upon GSK3β inhibition (top panel) and their neurosphere formation capacity in stem cell-specific medium (bottom panel). **c** Representative immunoblots of β-catenin and Gli1 showing dose-dependent increase upon GSK3β inhibition in U87MG, which was confirmed by decreased protein level of phospho-GSK3β Ser9 and total GSK3β. **d** Representative phase-contrast images showing enhanced neurosphere formation in U87MG and iU87MG in the presence of GSK3β inhibitor. **e** Quantitative representation of number and size of neurospheres upon GSK3β inhibition in U87MG and iU87MG. Results represent mean ± SD from three independent experiments

So far, we have shown the involvement of β-catenin and Gli1 in the dedifferentiation of U87MG to GSCs upon NS induction. Stabilization of β-catenin and Gli1 is mediated by the GSK3β activity. Phosphorylation at Ser473 is an activatory phosphorylation of AKT, which is an immediate upstream kinase of GSK3β for Ser9 phosphorylation that inhibits its activity. We observed higher phosphorylation at Ser9 of GSK3β, and Ser473 of AKT in iU87MG, suggesting their better survivability controlled by reduced GSK3β activity (Fig. 5a).

Next, we checked the role of deactivation of GSK3β in such dedifferentiation under NS. Therefore, U87MG was cultured in the presence of a GSK3β inhibitor (SB-216763), and subsequently subjected to NS for 5 days. U87MG pre-treated with an inhibitor showed moderately larger neurosphere-like phenotype, and a higher concentration of SB-216763 was more efficient for this phenotypic transition from U87MG to iU87MG (Fig. 5b, top panel). Treated iU87MG exhibited increased β-catenin and Gli1 expression compared to untreated iU87MG (Fig. 5c). GSK3β inhibition was validated by the reduction of total protein, and its Ser9 phosphorylation. Moreover, when GSK3β-inhibited iU87MG was further cultured in a neurosphere-forming medium, only a marginal increase in the number of neurospheres was observed, as GSK3β activity is already reduced in these cells (Fig. 5b, bottom panel).

Furthermore, both U87MG and iU87MG were treated with SB-216763 separately, and cultured in neurosphere-forming medium. They formed higher and larger neurospheres than untreated cells (Fig. 5d, e). Interestingly, fold increase in size and number of neurospheres was also significant in U87MG when GSK3β activity is inhibited. This indicated that deactivation of GSK3β activity plays an important role for NS-induced dedifferentiation, and this is a dominant feature in iU87MG.

### Loss-of-function mutation of PTEN is a central regulator of NS-induced dedifferentiation

PTEN is usually inactivated in GBM, and we established a cellular cross-talk between PTEN and mTORC2 via Rictor^27^. Here, we addressed the role of PTEN in NS-induced dedifferentiation of GBM cells.

**Fig. 6:**
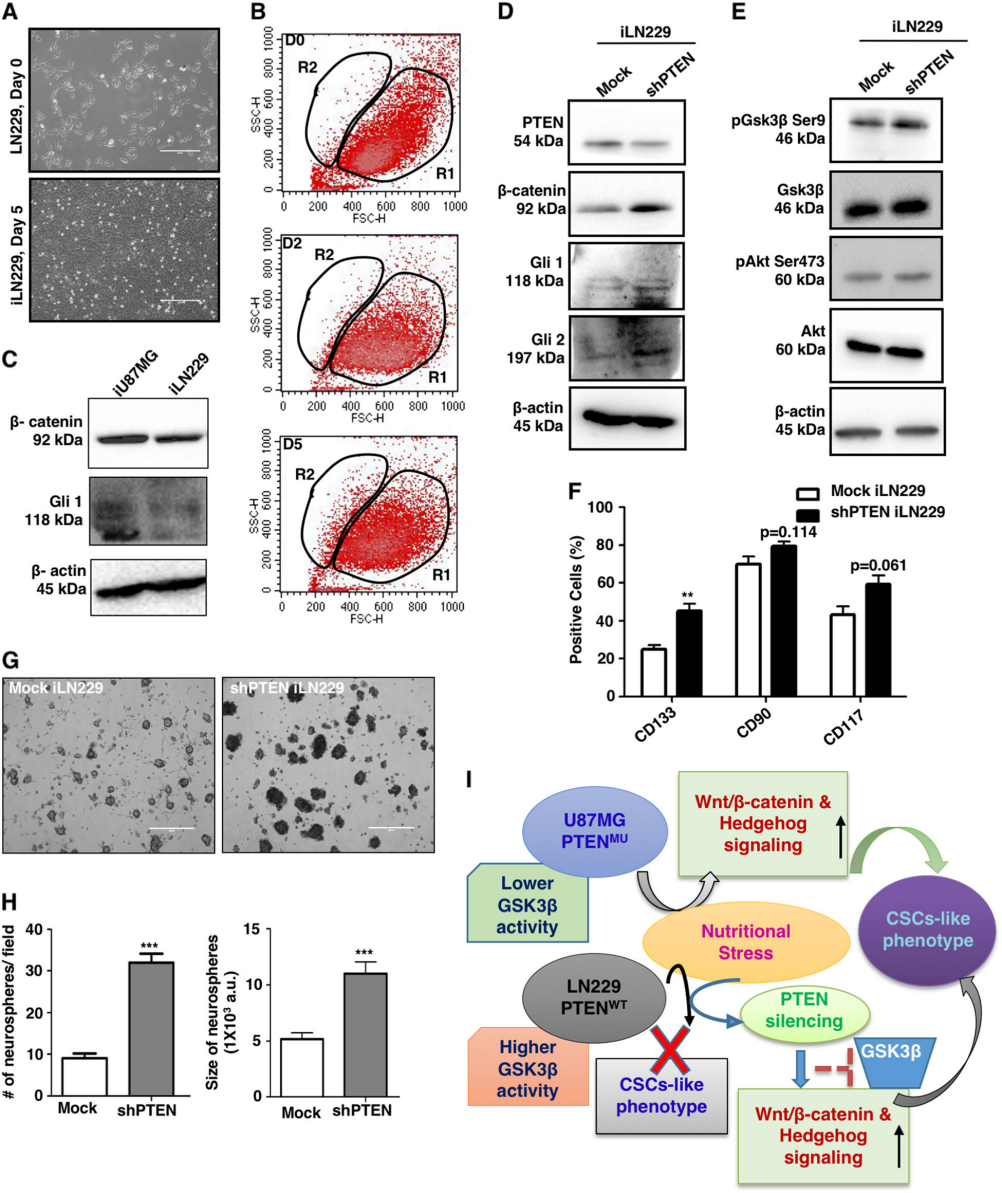
Role of PTEN in the regulation of stemness property in NS-induced cells. **a** Representative phase-contrast images of PTEN^wt^ LN229 after 5 days of NS induction (iLN229) showing no sphere-like morphology. **b** Flow cytometric analysis showed no significant shifting of iLN229 toward smaller-sized R2 population after day 2 (D2) and day 5 (D5). **c** Western blot analysis showed a lower level of β-catenin and Gli1 in iLN229 (PTEN^wt^) compared to iU87MG (PTEN^mu^). **d** LN229 (5 × 10^5^) cells were transfected with PTEN shRNA using Lipofectamine LTX and Plus reagents followed by 5 days of nutritional stress. Representative immunoblots showing an increased level of β-catenin, Gli1, and Gli2 in PTEN-knockdown iLN229. **e** PTEN-knockdown iLN229 showed higher GSK3β phosphorylation at the Ser9 position, leading to lower GSK3β activity compared to mock-transfected iLN229, which is mediated by higher AKT phosphorylation at Ser473. **f** PTEN-knockdown iLN229 showed higher expression of GSCs markers compared to mock-transfected iLN229 as assessed by flow cytometry. **g** Representative phase-contrast images showing larger neurosphere formation in PTEN-knockdown iLN229 compared to mock-transfected iLN229. **h** Quantitative representation of number and size of neurospheres in PTEN-knockdown iLN229. **i** Schematic representation of nutritional stress-mediated emergence of GSCs by modulating Wnt and Hedgehog signaling and involvement of PTEN. Results represent mean ± SD from three independent experiments

We compared dedifferentiation of PTEN^mu^ (U87MG) with PTEN^wt^ (LN229) cells under similar metabolic stress. No striking morphological transitions in PTEN^wt^ cells even after 5 days of NS were observed (Fig. 6a) as in U87MG (Fig. 1b). Compared to Fig. 1c, no distinct R2 population was found in PTEN^wt^ cells (Fig. 6b).

So far, we have established that both β-catenin and Gli1 are involved in dedifferentiation of GBM under NS (Fig. 4). Therefore, we compared the status of β-catenin and Gli1 in PTEN^mu^ and PTEN^wt^ cells under NS, and a significantly lower expressions of β-catenin, Gli1 and Gli2 in PTEN^wt^ cells are observed (Fig. 6c).

To establish the role of PTEN, we knocked it down by using short hairpin RNA (shRNA) in LN229 cells, subsequently subject to NS for 5 days (iLN229), and monitor their ability to form neurospheres. PTEN-knocked down iLN229 exhibited higher expression of β-catenin, Gli1 and Gli2 compared to mock-transfected iLN229 (Fig. 6d).

To confirm these observations, we checked the phosphorylation levels of GSK3β at Ser9 and AKT at Ser473. There were increased activatory phosphorylation of AKT and inhibitory phosphorylation of GSK3β in PTEN-knocked down iLN229 (Fig. 6e).

This PTEN-knocked down iLN229 also showed enhanced CD133, CD90 and CD117 expressions than PTEN^wt^ iLN229 after 5 days of NS induction, as observed in PTEN^mu^ iU87MG (Fig. 6f).

Furthermore, this PTEN-knocked down iLN229 exhibited significantly higher neurosphere formation potential according to the size and number of the sphere, like iU87MG (Fig. 6g, h). Collectively, PTEN behaves like a master regulator of NS-induced GBM cells dedifferentiation by reducing GSK3β activity, and subsequent enhancement of β-catenin/Gli regulations (Fig. 6i).

## Discussion

Tumor microenvironment provides metabolic challenges to CSCs^28^. Although the role of hypoxic stress in tumor progression is known^29,30^, the role of micro-environmental NS in dedifferentiation of cancer cells, especially in the emergence and maintenance of GSCs, is still poorly defined in GBM. Here, we demonstrated the role of micro-environmental NS in the regulation of GSCs.

Accordingly, we provided a cell culture system to mimic the continually changing microenvironment of a propagating tumor. Our system resulted in a gradual nutrient deprivation, lowering of pH, growth-space crisis and hypoxic core. Such combinations drive the stochastic stress adaptation of cancer cells, and induce GSC-like features. Thus, we have generated a simple stochastic CSC model system, and validated its morphological and functional characteristics. Our findings establish that gradual changes in tumor microenvironment force cancer cells to undergo dedifferentiation pathways, and stochastically change the cellular state in favor of cancer.

Thus, the major achievement of our study is to develop a stochastic model of CSCs by gradual nutritional deprivation which mimics the tumor microenvironment representing the core of tumor mass. Induction of such NS allows GBM to stochastically adapt to the microenvironment so that it gains the higher capacity of neurosphere formation, GSC-specific biomarkers expressions with enhanced invasive, angiogenic and migratory properties. Drug resistance of CSCs is the main challenge for efficient cancer therapy^31^. This NS-induced cells showed superior xenobiotic efflux ability and hence exhibit resistance to multiple anticancer drugs. In summary, our in vitro system allowed us to uncover the role of micro-environmental NS in mediating the stochastic emergence of GSCs.

Additionally, at the molecular level, we have demonstrated enhancement of molecules related to stem cell regulatory-Wnt/β-catenin and Hh pathways by stabilizing β-catenin/Gli1 through modulation of GSK3β/AKT axis, confirming again the involvement of NS in the maintenance of GSCs.

More importantly, we have established that PTEN mutation contributed better phenoconversion of stochastic adaptation of GBM cells toward GSC phenotypes. Knocking down of PTEN coupled with NS induction enhanced neurosphere formation, GSC-specific biomarker expressions, and activation of Wnt/Hh signaling.

The vital prerequisites of GSCs are to form neurospheres and clonal proliferation in tumor niche. Here, we hypothesize that NS may be a critical factor to switch on a program for a visible transition of differentiated cells toward dedifferentiated neurosphere-like population.

In adult tissues, primitive quiescent stem cells are smaller in size than their differentiated progeny^32^. We also observed the transition of the cell size from larger to smaller (R1 to R2) over a period of time during NS induction, which correlates with our hypothesis of NS-induced dedifferentiation and formation of a newly transformed smaller-sized cell population (R2), indicative of GSCs.

This smaller cell-sized R2 population also displayed higher GSC markers, suggesting a positive correlation between smaller cell size and higher stemness properties. Additionally, we also observed an overall increase in the GSC marker expressions in the total population, suggesting stochastic adaptability of the differentiated cells toward dedifferentiated phenotype under NS.

Consistent MFI values of these GSC markers in the total (R1+R2) population throughout NS induction signify that each non-stem cell has a potential to become a stem cell, again confirming the stochastic adaptability. Therefore, cells possessing higher stemness properties residing in the R2 population are already present in the heterogeneous cancer cell population, and they are not the only candidate to become GSCs.

Higher expressions and nuclear localizations of stem cell-specific transcription factors were also observed under NS induction, which may be responsible for the maintenance of stem cell-like properties by the modulation of several downstream pathways in GBM.

These NS-induced neurospheres exhibited GSC-like functional properties with higher expression of VEGF, Slug and Snail accompanied by a higher rate of neurosphere formation, superior invading, migrating properties and connective tube formation.

Another important characteristic of CSCs is their quiescent nature in cell cycle progression. NS-induced cells demonstrated G0/G1 cell cycle restriction, and accumulation of Cyclin D, Cyclin D2 and Cyclin E, necessary molecules for the transition to the S phase, suggesting that these GSCs are ready for re-differentiation under favored condition. Indeed, we observed that re-seeding of NS-induced GSCs with fresh growth medium and subsequent medium replenishment ensured re-differentiation by the morphological transition to epithelial nature, and a higher number of cells in the S phase compared to dedifferentiated GSCs, indicating the bracing of these quiescent NS-induced cells.

GBM is resistant against temozolomide, paclitaxel, 5-fluorouracil and cisplatin, due to the extensive activities and expressions of drug efflux pumps and DNA damage repair system. NS induction influenced the drug efflux mechanism which leads to higher chemoresistance of these stress-induced GSCs.

One of the hallmarks of cancer cells is altered energy metabolism^33,34^. Preferential dependency on glycolysis is the main characteristic feature of the altered metabolic phenotype. NS-induced cells were cultured in the absence of different major metabolic factors needed for glycolysis and oxidative phosphorylation such as glucose, pyruvate, and even fetal bovine serum (FBS) to understand the importance of the altered physicochemical milieu in cancer for the stress adaptability. Differential metabolic needs were observed for NS-induced GSCs. We noticed a strong glycolysis dependency of GBM cells, and its NS-induced derivatives. Moreover, NS-induced GSCs were also resistant toward these additional metabolic stresses showing their efficient stress-adaptive nature. Under hypoxic condition, glycolysis becomes the main energy metabolism pathway for cancer cells. Hypoxic microenvironment promotes CSCs generation and maintenance^35^. NS alone stabilized HIF-1α, thereby promoting GSCs-like features, and additional hypoxia induction further enhanced the stochastic adaptability of GSCs. Our culture system provided a gradual metabolic deprivation as well as oxygen deprivation in the miniature tumor spheres, which leads to the phenoconversion of GBM cells to GSCs.

CSCs share many characteristics of embryonic stem and tissue progenitor cells. CSCs often display activation of highly conserved developmental, and tissue homeostatic and regeneration mechanisms such as Wnt, Hh and Notch signaling pathways^36^. Therefore, the development of therapeutic strategies targeting these pathways is important. Our findings demonstrated that micro-environmental stresses encourage activation of Wnt and Hh pathways, thereby stirring GBM cells to reprogram their developmental features. Inhibitions of Wnt and Hh pathways attenuated neurosphere formation, suggesting that proliferation and maintenance of NS-induced GSCs rely on these pathways.

The key transcription regulators of Wnt and Hh pathways (β-catenin and Gli1) are negatively regulated by GSK3β and CKI kinases^37^. Here, we also observed the higher inactivation of GSK3β in NS-induced GSCs, suggesting that GSK3β inhibition, in turn, results in the larger neurosphere-like morphology, and exhibited higher activities and expression of β-catenin and Gli1. Moreover, GSK3β inhibition facilitated better neurosphere formation in U87MG, which is a feature of NS-induced cells.

We have earlier established a positive correlation between PTEN mutations and mTORC2 hyperactivity, which negatively regulates GSK3β activity in GBM, and a connection between mTORC2 and Hh pathway in the context of GSK3β activity^38^. As GSK3β is turning out to be a functional mediator of this NS-induced stemness, we assessed the role of PTEN in this context. We observed that functional loss of PTEN provides better stress adaptability, and change of cellular state to stem-like cells. Genetic depletion of PTEN in PTEN^wt^ cells ensured higher GSC marker expression and neurosphere formation, indicating better adaptability and dedifferentiation upon nutritional or metabolic stress conditions.

Taken together, we provide evidences for a novel role of micro-environmental NS in promoting the emergence of CSCs by modulating Wnt and Hh signaling pathways. In summary, our study has opened up a new avenue for designing a modified GSCs culture system, which would be helpful for understanding GSCs regulations, and uncovering different therapeutic modalities under such mimicked conditions. Therefore, such an in-depth understanding of dedifferentiation of GBM cells to GSCs under NS suggested that targeting Wnt/Hh signaling possibly be a better anti-cancer therapeutic approach.

## Materials and methods

### Reagents

Iscove’s modified Dulbecco’s medium (IMDM) and FBS were from Gibco, Life Technologies (Carlsbad, CA, USA); Lipofectamine LTX, Plus reagents, Matrigel, DMEM/F12, and B27 supplement were from Invitrogen (Carlsbad, CA, USA). Epidermal growth factor (EGF), basic fibroblast growth factor (bFGF), heparin, temozolomide, paclitaxel, 5-FU, cisplatin, SB-216763, and IWR-1 were from Sigma-Aldrich. Antibodies for Oct-4A (2840), Sox2 (3579), Nanog (4903), VEGF (2463S), Slug (9585S), Snail (3879S), Cyclin D1 (2978S), Cyclin D2 (3741S), Cyclin E (81045S), HIF-1α (36169), HIF-1β (5537), Frizzled 5 (5266), Wnt5a/b (2530S), Dvl2 (3324), Dvl3 (3218), LRP6 (3395), Nkd1 (2201), β-catenin (8480), Gli1 (3538S), Sufu (2522S), Shh (2207S), GSK3β (9315), phospho-GSK3β-Ser9 (5558), Akt (4691), phospho-Akt-Ser473 (4060), β-actin (4970), α-tubulin (2125S), HDAC3 (2632S), rabbit IgG (3900S), horse radish peroxidase (HRP)-conjugated anti-rabbit antibodies (7047S), and anti-mouse secondary antibodies (7076) were from Cell Signaling Technology (Danvers, MA, USA). Gli2 (sc-28674) and Wnt2 (sc-514382) antibodies, and GANT-61 were from Santa Cruz Biotechnology (Santa Cruz, CA, USA).

### Cell culture

U87MG and LN229 human GBM cells (ATCC, Manassas, VA, USA) were cultured in IMDM (Invitrogen) with 10% FBS in a humidified 5% CO_2_ incubator at 37 °C. For inducing NS, cells (5 × 10^5^ cells/well) were placed in a six-well plate in IMDM (2 ml) with 10% FBS and cultured for 5 days without replenishment of fresh medium.

For generating a neurosphere, cells (1 × 10^5^) were seeded on 12-well low-attachment Petri dishes in serum-free neural stem cell medium composed of DMEM/F12, 20 ng/ml bFGF, 20 ng/ml EGF, 10 ng/ml heparin, and B27 supplement. Neurospheres were photographed and counted from three randomly selected fields using light-inverted microscopy (Life Technologies). Alternatively, cells (1 × 10^4^−1 × 10^6^) were also treated with various inhibitors under similar conditions for specific experiments.

For specific metabolic depletion, U87MG and NS-induced cells (iU87MG, 1 × 10^5^) were cultured separately for an additional 72 h in absence of serum, glucose and pyruvate alone, and glucose and pyruvate together . For the induction of hypoxic stress, U87MG and iU87MG cells were incubated with cobalt chloride (CoCl_2_, 100 µM) for 24 h in the CO_2_ incubator.

### Flow cytometry

Both U87MG and iU87MG cells were made from a single-cell suspension, incubated for 15 min with anti-CD133-APC, anti-CD90-PECy5, and anti-CD117-PE (BD PharMingen) as per the manufacturer’s instructions. Samples were acquired using FACSCalibur and analyzed by Cell Quest Pro software. Side and forward scatter were used to distinguish the cell sizes and further analyzed for the expression of CD133, CD90, and CD117^39^. To check the viability of the iU87MG cells, they were stained with PI using the manufacturer’s protocol (BD Biosciences).

After metabolic depletion, cells were processed using the Cell Cycle Test Plus Kit (BD Biosciences). Data acquisitions were accomplished using FACS LSRFortessa and data were analyzed using FACSDiva 8.0.2 software.

For determination of side population, U87MG and iU87MG (1 × 10^7^/ml) cells were suspended in complete medium with Hoechst 33342 and incubated at 37 °C for 2 h. Additionally, cells were incubated with PI according to the manufacturer’s protocol to eliminate the dead cell population. Data acquisitions were accomplished using FACS LSRFortessa and analyzed using FlowJo v8.7 software.

### qRT-PCR analysis

Total cellular RNA was isolated using the RNeasy Kit and quantified using a NanoDrop spectrophotometer. Complementary DNA was synthesized with random primers (Supplementary Table S1) using the ImProm-II RT system (Promega, USA). Real-time PCR was performed using a DyNAmo Flash SYBR Green qPCR Kit. Relative amounts of target mRNAs were quantified using the LightCycler 96 (Roche) software with 18S ribosomal RNA (rRNA) as an internal control.

### Immunoblotting

Cells were sonicated (Qsonica-LLC, XL-2000) in ice-cold phosphate-buffered saline (PBS) and proteins were estimated using the BCA Assay Kit (Thermo Scientific). Cytosol and nucleus were separated using a standard protocol. Equal amounts of proteins (40–80 µg) were separated by sodium dodecyl sulfate-polyacrylamide gel electrophoresis (5–12%) and electrotransferred to nitrocellulose membrane. The membrane was blocked with Tris-buffered saline (TBS)–bovine serum albumin (2–5%) for 5–30 min and probed with the primary antibody. Blots were washed with TBS–Tween-20, incubated with HRP-conjugated secondary antibodies, and detected by West-pico ECL system. Images were captured by Bio-Rad ChemiDoc MP and analyzed with Image Lab software version 5.2.1.

### Immunocytochemical staining

U87MG and iU87MG (1 × 10^4^) cells were adhered on poly-l-lysine-coated coverslip for 3 h, washed with PBS-0.3% Tween-20 (PBS-T), and fixed with paraformaldehyde. Cells were incubated with goat serum (1 h, 25 °C) and then with primary antibodies overnight at 4 °C followed by incubation with Alexa Flour 488-conjugated secondary antibodies for 3 h at 25 °C. Next, the cells were further stained with DAPI (4′,6-diamidino-2-phenylindole) for 10 min, washed, and then mounted. Cells were visualized using the confocal microscope. Representative merged images of Alexa Flour 488 and DAPI were shown in the figures.

### Invasion assay

U87MG and iU87MG (5 × 10^4^) cells were added to the upper part of Matrigel-coated invasion chamber in serum-free medium (200 µl) and the lower chamber was filled with the medium with FBS (600 µl). The cells on the lower surface of the insert were stained with the crystal violet after 36 h and counted by light-inverted microscopy.

### Scratch-wound assay

U87MG and iU87MG cells were harvested in six-well plates with >90% confluence. Scratch wounds were made by a 10-µl micropipette tip, washed thrice, and incubated in the incubator with IMDM and 1% FBS to inhibit the cell proliferation. Images were captured at 0, 8, and 24 h. Wound size was calculated using ImageJ software and represented as a bar graph.

### Connective tube formation

U87MG and iU87MG (5 × 10^4^) cells were seeded onto the Matrigel-coated 24-well plate and incubated for 72 h in the CO_2_ incubator. Images were captured from randomly selected fields as above.

### Cell viability assay

U87MG and iU87MG (1 × 10^4^/250 µl per well) cells were incubated with different concentrations of paclitaxel, temozolomide, 5-fluorouracil, and cisplatin for 24 h and cell death was determined by the MTT (3-[4,5-dimethylthiazol-2-yl]-2,5-diphenyl tetrazolium bromide) assay.

### Transient transfection

LN229 (5 × 10^5^) cells were transfected with PTEN shRNA (Addgene, plasmid#10669, 2 μg/ml) using Lipofectamine LTX and Plus reagents. Plasmid constructs were amplified and isolated using Qiaprep Spin Miniprep Kit (Qiagen).

### Luciferase reporter assay

Regulation of Gli transcription was measured using a firefly luciferase-based Gli-Reporter Assay (8GLI-luciferase, Signal reporter Gli) Kit. U87MG and iU87MG cells were transfected with negative and positive controls, Gli reporter. To measure β-catenin activity, cells were transfected with either Super8TOPFlash plasmid (#12456) or Super8FOPFlash (#12457) reporter constructs (Addgene) which were kind gifts from Prof. Randall Moon, University of Washington. Cells were processed using the Promega Luciferase Kit after 48 h.

### Statistical analysis

All the data were from at least three independent experiments and statistical analysis was performed using GraphPad Prism 5. The differences between the groups were analyzed by two-tailed Student’s *t* test or Mann–Whitney *U* test. Standard error bars represent the standard deviation of the mean (±SD) and *P < 0.05, **P < 0.01, and ***P < 0.001 denoted the significant differences between the means of the two test groups.

## Electronic supplementary material

Supplementary figure
