# Supplementary material for: Nutritional stress reprograms dedifferention in glioblastoma multiforme driven by PTEN/Wnt/Hedgehog axis: a stochastic model of cancer stem cells
Source: Cell Death Discov. 2018 Dec 5;4:110. doi: 10.1038/s41420-018-0126-6 (PMC6281623; doi:10.1038/s41420-018-0126-6)

Figure S1

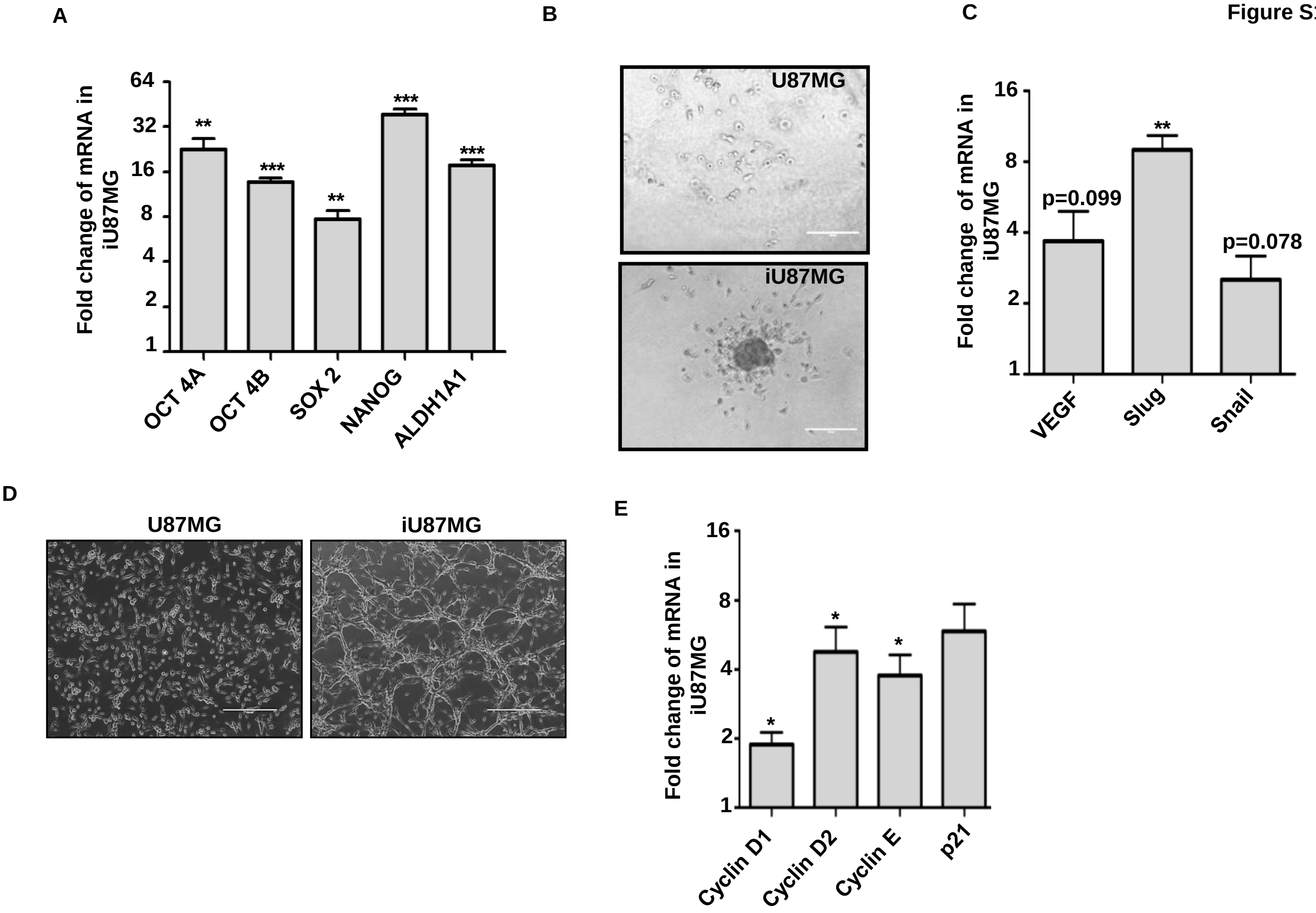

Figure S1. Phenotypic characteristics of iU87MG cells (related to Figure 2)

A. Expression of pluripotent stem cell markers and ALDH1A at mRNA level in iU87MG compared to U87MG as assesses by real-time qPCR. Values are normalized against 18S rRNA expression.

B. Representative image of a 3D-sphere formed by iU87MG in a matrigel-coated plate after 72 hrs.

C. Fold changes in the mRNA level of Slug, Snail and VEGF in iU87MG over U87MG as assesses by real-time qPCR. Values are normalized against 18S rRNA.

D. iU87MG showed higher connective tube formation after 72hrs on top of a matrigel coated plate.

E. Accumulation of Cyclin D1, Cyclin D2, CyclinE and p21 at mRNA level in iU87MG over U87MG as assesses by real-time qPCR. Values are normalized against 18S rRNA expression.

Figure S2

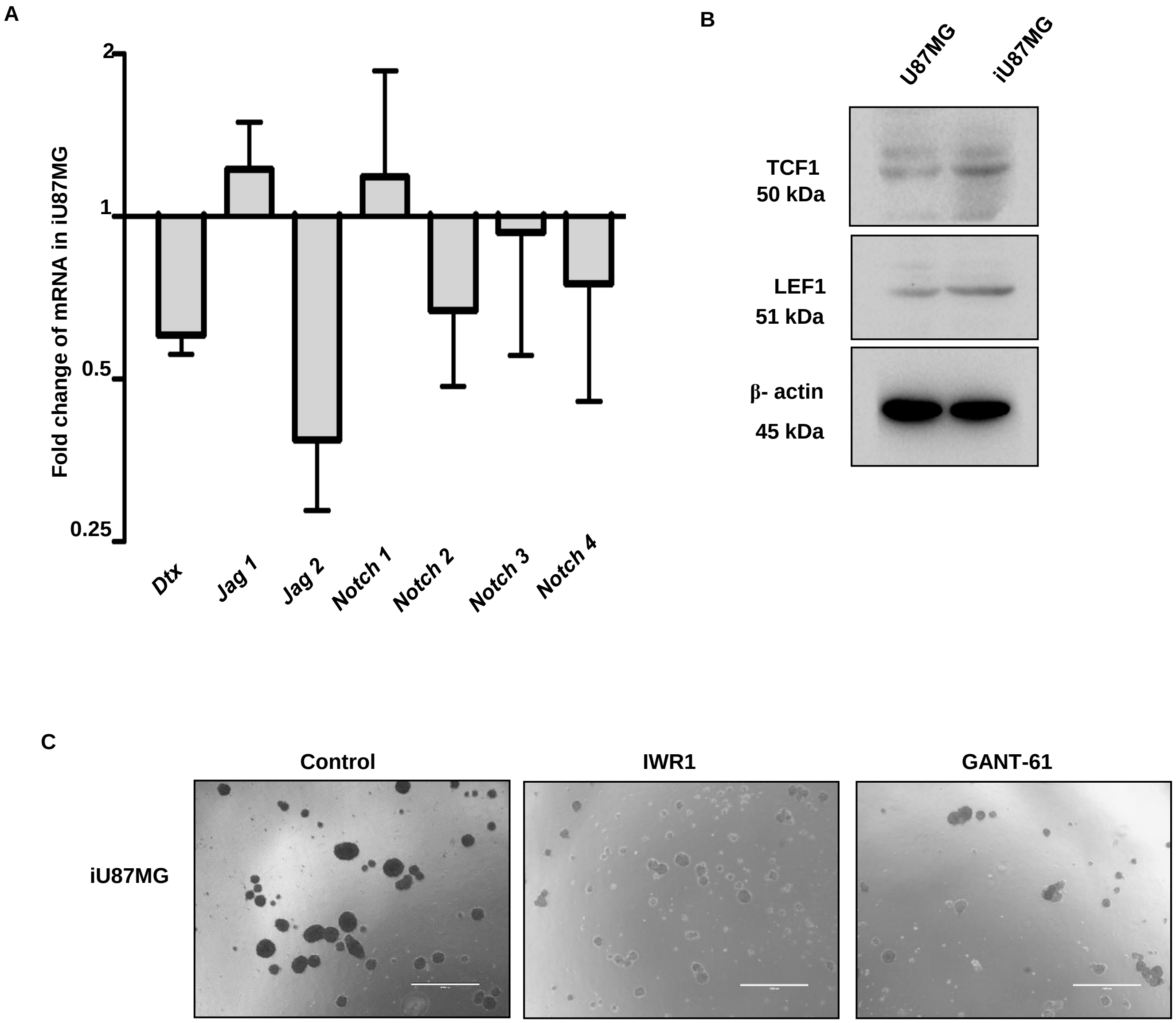

Figure S2. Involvement of stem cells regulatory pathways in NS-induced cells (related to Figure 4)

A. mRNA expression of Notch pathway genes showed down regulation in iU87MG compared to U87MG.

B.Western blots of target genes of β-catenin and Gli1showed enhancement upon nutritional stress.

C. Phase contrast images showed neurospheres formation ability of pre-treated iU87MG with Wnt/β-catenin and Sonic Hedgehog pathway inhibitors (IWR1 and GANT61 respectively).

Supplementary Table S1: List of primers details

NAME	FORWARD PRIMER	REVERSE PRIMER
OCT 4A	GAGGAGTCCCAGGACATCAA	ACACTCGGACCACATCCTTC
OCT 4B	GAAGTTAGGTGGGCAGCTTG	CTCCAGGTTGCCTCTCACTC
SOX 2	AGAACCCCAAGATGCACAAC	ATGTAGGTCTGCGAGCTGGT
NANOG	ACCAGACCCAGAACATCCAG	CTCGTCGATTAGGCTCCAAC
ALDH 1A	TGGAGTCAATGAATGGTGGA	CAAGTCGGCATCAGCTAACA
CYCLIN D1	CCTAAGTTCGGTTCCGATGA	ACGTCAGCCTCCACACTCTT
CYCLIN D2	ATTGGCATGTCTGGTTCACA	GCCAGATACCAGAAGCGAAG
CYCLIN E	AGCGGTAAGAAGCAGAGCAG	TTTGATGCCATCCACAGAAA
P21	ATGAAATTCACCCCCTTTCC	CCCTAGGTCGTGCTCACTTC
VEGFA	AGGCCAGCACATAGGAGAGA	TTTCTTGCGCTTTCGTTTTT
SNAIL	GAGGACAGTGGGAAAGGCTC	TGGCTTCGGATGTGCATCTT
SLUG	CATCTTTGGGGCGAGTGAGT	GGCCAGCCCAGAAAAAGTTG
WNT 2	GTGGATGCAAAGGAAAGGAA	AGCCAGCATGTCCTGAGAGT
FRIZZLED 7	GTACTTTAAGGAGGAGGAGA	GTAGGTGAGAACGGTAAAGA
DISHEVELED 2	TGAGAGCTACCTAGTCAACC	GGGTATTGGTAGGAGAAAGT
DISHEVELED 3	GAGACCAAGATCATCTACCA	CGTCCATAGACTTGAAGAAG
AXIN 1	AGAGCCATCTACCGAAAGTA	ACTTAAGGAAGGAGGGATAG
AXIN 2	CATAGGTTCTGGCTATGTCT	TCTCTGGAGCTGTTTCTTAC
LRP 6	TTGTGACAGTGACTATGCTC	TCTCTGTGTATGGAGAAGGT
β- CATENIN	TCCTTCTCTGAGTGGTAAAG	CATCTAATGTCTCAGGGAAC
GLI1	GTGCAAGTCAAGCCAGAACA	ATAGGGGCCTGACTGGAGAT
GLI2	TTTATGGGCATCCTCTCTGG	AAGGCTGGAAAGCACTGTGT
PTCH1	TCCCAGCGCTTTCTACATCT	CTTTGTCGTGGACCCATTCT
SHH	TACTCGCAGCTGCTCTACCA	TGTCTTTTTGCTTTGCGTTG
SMO	CAACCTGTTTGCCATGTTTG	TTTGGCTCATCGTCACTCTG
JAGGED 1	GAGATGACTTCTTTGGACAC	TGCACTTATCACAGTACAGG
JAGGED 2	CTGCGACGAGAACTACTACA	CTTGTTTACACACAGCTTCC
NOTCH 1	AGTACTGTACCGAGGATGTG	GCACAGTCATCAATGTTCTC
NOTCH 2	ACTGCCAGTACTCAACATCT	GGTACAGGTACTTCCATTTG
NOTCH 3	AGAGCTGCAGTCAGAATATC	GTGTCACAGATAGCATCCTC
NOTCH 4	TAGTGAGGAGATGACAGCTT	ACAGTAGTCAGTGCTGGTTT
C-MYC	GTCAGAGTCTGGATCACCTT	ACTCTGACACTGTCCAACTT
MMP-2	GGACACACTAAAGAAGATGC	CCTGTATGTGATCTGGTTCT
MMP-7	CTTTGGCCTACCTATAACTG	ATGATACGATCCTGTAGGTG
α-CATENIN	GCTGTCTCAGAGAATCACAT	TCCATCTCTGAGGTGACTAC
TCF4	AGAGCTGAGTGATTTACTGG	CCTGAGCTACTTCTGTCTTC
LEF1	AGCTCCTGATATCCCTACTT	GTTACAATAGCTGGATGAGG

Figure S2
For Reviewer only

Full scanned images of blots of Nestin Figure 2A

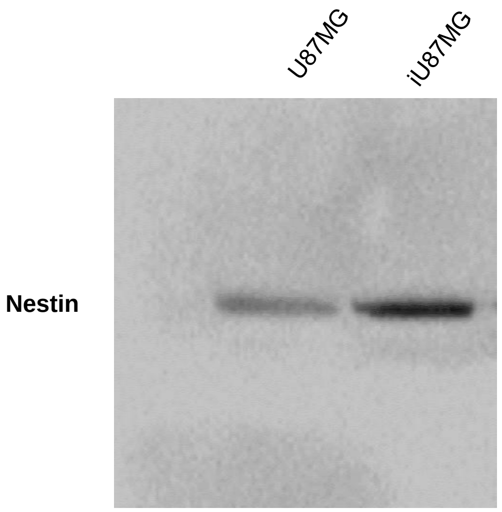

Supplement: Supplementary file 1 — Supplementary figure [file 41420_2018_126_MOESM1_ESM.pdf]
